# Effect of disconnecting the orbital prefrontal cortex from the nucleus accumbens core on inter-temporal choice behaviour: A quantitative analysis

**DOI:** 10.1016/j.bbr.2008.03.041

**Published:** 2008-08-22

**Authors:** G. Bezzina, S. Body, T.H.C. Cheung, C.L. Hampson, C.M. Bradshaw, E. Szabadi, I.M. Anderson, J.F.W. Deakin

**Affiliations:** a Psychopharmacology Section, Division of Psychiatry, University of Nottingham, Room B109, Medical School, Queen's Medical Centre, Nottingham NG7 2UH, United Kingdom; b Neuroscience & Psychiatry Unit, School of Psychiatry & Behavioural Sciences, University of Manchester, Stopford Building, Oxford Road, Manchester M13 9PT, UK

**Keywords:** Orbital prefrontal cortex, Nucleus accumbens core, Disconnection lesion, Inter-temporal choice, Delay discounting, Rat

## Abstract

Previous experiments showed that destruction of the orbital prefrontal cortex (OPFC) or the nucleus accumbens core (AcbC) in rats altered choice between two delayed food reinforcers. Application of a quantitative model of inter-temporal choice suggested that lesions of either structure increased the delay-dependent degradation of reinforcer value (delay discounting); destruction of the OPFC (but not the AcbC) also increased the relative value of the larger reinforcer. This experiment examined the effect of disconnecting the OPFC from the AcbC on inter-temporal choice. Rats received excitotoxin-induced contralateral lesions of the OPFC and AcbC (disconnection), severing of the anterior corpus callosum (callosotomy), a combined lesion (disconnection + callosotomy) or sham lesions. They were trained in a discrete-trials progressive delay schedule to press levers A and B for a sucrose solution. Responses on A delivered 50 μl of the solution after a delay *d*_A_; responses on B delivered 100 μl after a delay *d*_B_. *d*_B_ increased across blocks of trials; *d*_A_ was manipulated across phases of the experiment. Indifference delay, *d*_B(50)_ (value of *d*_B_ corresponding to 50% choice of B), was estimated for each rat in each phase, and linear indifference functions (*d*_B(50)_*vs. d*_A_) were derived. The disconnection + callosotomy group showed a lower intercept of the indifference function (implying a higher rate of delay discounting) than the sham-lesioned group; the disconnection group showed a similar but less robust effect, whereas the callosotomy group did not differ significantly from the sham-lesioned group. The results suggest that OPFC–AcbC connections are involved in delay discounting of food reinforcers, but provide no evidence for an involvement of OPFC–AcbC connections in regulating sensitivity to reinforcer size.

## 1 Introduction

It is widely accepted that the capacity of a reinforcer to exert control over operant behaviour is a direct function of its size and an inverse function of the delay that precedes its delivery. These two principles are placed in mutual opposition in inter-temporal choice schedules, in which the subject is required to choose between two reinforcers that differ along both dimensions. For example, the subject may be confronted with a choice between a smaller reinforcer, *A*, of size *q*_A_, delivered after a short delay, *d*_A_, and a larger reinforcer, *B*, of size *q*_B_, delivered after a longer delay, *d*_B_.

Inter-temporal choice schedules have provided valuable insights into the behavioural and neurobiological bases of ‘delay discounting’, the hypothetical process whereby the efficacy or ‘value’ of a reinforcer decays as a function of delay. Recent work has implicated the core of the nucleus accumbens (AcbC) [1,2,6,8] and the orbital region of the prefrontal cortex (OPFC) [23,26,42] in inter-temporal choice behaviour. However, the interpretation of results obtained using these schedules is complicated by the fact that magnitude and delay of reinforcement are generally manipulated simultaneously. Therefore it is often unclear whether the effect of an intervention on inter-temporal choice behaviour has been brought about by a change in the rate of delay discounting, by a change in the organism's sensitivity to relative reinforcer size, or both [8,19,31]. One approach to overcoming this problem is the application of null equations derived from quantitative models of inter-temporal choice [19,29–31]. For example, according to one such model [19], the overall value of a reinforcer, *V*, is determined by the multiplicative combination of two hyperbolic expressions that define the effects of delay and magnitude upon reinforcer value:

(1) $$V=\frac{1}{1+Kd}.\frac{1}{1+(Q/q)}\text{,}$$

where *K* is the delay-discounting parameter [29] and *Q* is a parameter expressing sensitivity to reinforcer size [19]. Faced with a choice between two reinforcers, A and B, the organism is presumed to select the reinforcer with the higher value. However, by experimentally manipulating the sizes and delays of the two reinforcers, it is possible to establish a state of ‘indifference’, in which A and B are selected with equal frequency. Indifference between A and B is taken to imply equality of value, i.e., *V*_A_ = *V*_B_. Using Eq. (1) to define *V*_A_ and *V*_B_ and solving for *d*_B(50)_ (the delay to reinforcer B at the point of indifference), yields the following null equation:

(2) $$d_{\text{B}(50)}=\frac{1}{K}.\frac{1/(1+(Q/q_{\text{B}}))-1(1+(Q/q_{\text{A}}))}{1/(1+(Q/q_{\text{A}}))}+d_{\text{A}}.\frac{1+(Q/q_{\text{A}})}{1+(Q/q_{\text{B}})}\text{,}$$

in which *d*_B(50)_ is linearly related to *d*_A_. By examining the effect of an intervention on this relation, it is possible to deduce whether the intervention has affected the organism's rate of delay discounting, its sensitivity to reinforcer size, or both. Since *K* makes no contribution to the slope of the function, a change in slope implies a change in sensitivity to reinforcer size, whereas a change in intercept in the absence of a concomitant change in slope implies a change in the rate of delay discounting [19].

Destruction of the OPFC was found to increase the slope of the linear indifference function, indicating that the lesion affected sensitivity to reinforcer size; the lack of a concomitant increase in the intercept suggested that the rate of delay discounting had also been increased [26] (see Section 4 for further explanation). In contrast, lesions of AcbC reduced the intercept without significantly altering the slope of the function, implying a selective effect on delay discounting [2]. While these observations implicate both the OPFC and the AcbC in delay discounting, they leave unanswered the question of whether the two structures play independent roles in delay discounting, or whether they contribute to an integrated mechanism regulating inter-temporal choice behaviour. The present experiment was intended to address this question: the effect of functional disconnection of the OPFC and AcbC on inter-temporal choice behaviour was examined using the linear indifference relation epitomised by Eq. (2).

The principal anatomical link between the OPFC and the AcbC is an ipsilateral excitatory glutamatergic corticofugal pathway, which is believed to comprise one link in a cortico-striato-thalamo-cortical circuit. Inhibitory γ-aminobutyric acid (GABA)ergic efferents from the AcbC project to the internal pallidum and substantia nigra pars reticulata. These structures in turn send inhibitory projections to diencephalic structures, whose excitatory corticopetal projections complete the circuit. In order to effect functional disconnection of the OPFC and AcbC without totally ablating either structure, a ‘disconnection lesion’ [12,14] was employed, which consisted of unilateral excitotoxic destruction of the OPFC in one hemisphere and of the AcbC in the other hemisphere. As recent evidence indicates that transcallosal fibres may make a significant functional contribution to cortico-striatal connections [9], the effects of callosotomy alone, and callosotomy combined with the disconnection lesion were also examined.

## 2 Methods

The experiment was carried out in accordance with UK Home Office regulations governing experiments on living animals.

### 2.1 Subjects

Sixty experimentally naive female Wistar rats (Charles River UK) approximately 4 months old and weighing 250–300 g at the start of the experiment were used. They were housed individually under a constant cycle of 12 h light and 12 h darkness (light on 06:00–18:00 h), and were maintained at 80% of their initial free-feeding body weights throughout the experiment by providing a limited amount of standard rodent diet after each experimental session. Tap water was freely available in the home cages.

### 2.2 Surgery

Anaesthesia was induced with halothane (4% in oxygen), and the rat positioned in a stereotaxic apparatus (David Kopf), with the upper incisor bar set 3.3 mm below the inter-aural line. Anaesthesia was maintained with 2% halothane in oxygen during surgery. Holes were drilled in the skull for introduction of a microinjection cannula or leucotome (see below). *Disconnection*: sixteen rats received unilateral lesions of the OPFC and the contralateral AcbC (the sides being counterbalanced across rats). The following coordinates (mm, measured from bregma) were used to locate the OPFC: site (i): AP +3.7, L ±1.2, DV −4.8; site (ii): AP +3.7, L ±2.8, DV −4.4. The coordinates for the AcbC were: AP +1.2, L ±1.8, V −7.1. Injections were given via a 0.3-mm diameter cannula connected by a polyethylene tube to a 10-μl Hamilton syringe. In each site, 0.5 μl of a 0.1-M solution of quinolinic acid (2,3-pyridinedicarboxylic acid) in phosphate-buffered 0.9% NaC1 (pH 7.0) was injected at a rate of 0.1 μl per 15 s. The cannula was left in position for 3 min after completion of the injection. *Callosotomy*: 15 rats underwent a midline leucotomy in order to sever the anterior corpus callosum. The leucotome, similar to that described by Gold et al. [17], was constructed from a 1-μl Hamilton microsyringe. A curved wire could be extruded from the tip of the syringe needle; when fully extended, the wire projected approximately 3 mm in the AP plane (i.e. at right angles to the needle). Two midline cuts were made: (i) the tip of the needle was positioned at AP 0.0, DV −5.0, the wire extruded in a rostral direction, and the tip slowly raised to DV −1.0; the wire was then retracted into the needle, and the needle was withdrawn from the brain; (ii) the tip of the needle was repositioned at AP +2.0, DV −4.0 and the procedure repeated. *Disconnection* + *callosotomy*: 15 rats underwent both the disconnection and callosotomy procedures described above. *Sham lesion*: fourteen rats underwent the same surgical procedures as the disconnection-lesioned group, except that the vehicle solution alone was injected into the target sites.

### 2.3 Apparatus

The rats were trained in standard operant conditioning chambers (CeNeS Ltd., Cambridge, UK) of internal dimensions 25 cm × 25 cm × 22 cm. One wall of the chamber contained a recess into which a peristaltic pump could deliver a 0.6 M sucrose solution. Two apertures situated 5 cm above and 2.5 cm to either side of the recess, through which motor-operated retractable levers could be inserted into the chamber. The levers could be depressed by a force of approximately 0.2 N. A 2.8-W lamp was mounted 2.5 cm above each lever; a third lamp was mounted 10 cm above the central recess. Six red light-emitting diodes were mounted in a row, 4 cm apart, 5 cm above the levers. The operant chamber was enclosed in a sound-attenuating chest; masking noise was generated by a rotary fan. An Acorn microcomputer programmed in Arachnid BASIC (CeNeS Ltd., Cambridge, UK), located in an adjoining room, controlled the schedules and recorded the behavioural data.

### 2.4 Behavioural training

Two weeks after surgery, the food-deprivation regimen was introduced and the rats were gradually reduced to 80% of their free-feeding body weights. They were then trained to press two levers (A and B) for sucrose reinforcement, and were exposed to a discrete-trials continuous reinforcement schedule in which the two levers were presented in random sequence for three sessions. After this initial training, they underwent daily training sessions under the discrete-trials delayed reinforcement schedule for the remainder of the experiment. Each experimental session consisted of six blocks of six trials, except in phases 4 and 5 when sessions consisted of five blocks. The trials were 90 s in duration, with the exception of phase 5, in which the duration was increased to 120 s in order to accommodate the long delay to reinforcement (see below). The six blocks were signalled by illumination of the six light-emitting diodes: in block 1 the first (left-most) diode was illuminated, in block 2 the first and second diodes were illuminated, and so on. The first two trials of each block were forced-choice trials in which each lever was presented alone in random sequence. The other four trials were free-choice trials in which both levers were presented. The beginning of each trial was signalled by illumination of the central light above the reinforcer recess. After 2.5 s the lever or levers (depending on the type of trial) were inserted into the chamber. When a lever-press occurred, the lever(s) were withdrawn, the central light was extinguished, and the light located above the lever that had been depressed was illuminated. This light remained illuminated until the delivery of the reinforcer, and was then extinguished. The chamber remained in darkness until the start of the following trial. If no lever-press occurred within 5 s of the lever(s) being inserted, the lever(s) were retracted and the central light extinguished. (This seldom happened except during the first few training sessions.) A response on lever A initiated a fixed delay *d*_A_, following which 50 μl of the 0.6 M sucrose solution was delivered. A response on lever B initiated a variable delay *d*_B_, after which 100 μl of the same sucrose solution was delivered. The positions of levers A and B (left *vs.* right) were counterbalanced across subjects.

The experiment consisted of six phases, in which the value of *d*_A_ was set at 1, 2, 4, 8, 12 and 0.5 s, respectively. In each phase, the value of *d*_A_ was held constant. In each session the value of *d*_B_ was set equal to *d*_A_ in the first block of trials. In subsequent blocks *d*_B_ was increased in increments of 75%. In phases 4 and 5, when *d*_A_ was 8 and 12 s, respectively, computing five increments of 75% would have generated a value of *d*_B_ that was longer than the duration of a trial in the sixth block of trials; therefore the number of blocks was limited to five in these phases. The first phase continued for 100 sessions, phase 2 for 50 sessions, and the remaining phases for 40 sessions.

Experimental sessions were carried out 7 days a week, at the same time each day, during the light phase of the daily cycle (between 08:00 and 14:00 h).

### 2.5 Histology

At the end of the behavioural experiment, the rats were deeply anaesthetised with sodium pentobarbitone, and perfused transcardially with 0.9% sodium chloride, followed by 10% formol saline. The brains were removed from the skulls and fixed in formol saline for 1 week. Forty micrometer coronal sections were taken through the regions of the OPFC and AcbC (approximately from AP +5.0 to AP 0.0) using a freezing microtome.

#### 2.5.1 Cresyl violet staining

The procedure was similar to that described previously [26]. Alternate sections were mounted on chrome-gelatine-coated slides and air dried, hydrated by successive immersion in 95, 70 and 50% ethanol, stained in 0.25% cresyl violet for 2 min at room temperature, dehydrated by successive immersion in 50, 70, 95, and 100% ethanol and xylene, and mounted with DPX.

#### 2.5.2 Immunocytochemistry

In the other sections neurone-specific nuclear protein (NeuN) was labelled as described by Jongen-Relo and Feldon [21]. Our protocol has been described elsewhere [2]. Briefly, freshly sliced sections were rinsed in 0.1 M phosphate buffered saline (PBS) and placed in 0.5% H_2_O_2_ in PBS for 30 min. After twice rinsing in PBS, they were placed for 1 h in a blocking solution (10% normal horse serum [Vector Laboratories, Peterborough, UK], 1% bovine serum albumin [BSA, Sigma–Aldrich, Gillingham, UK] and 0.3% Triton X-100 [Sigma–Aldrich] in PBS). They were incubated for 48 h at 4 °C with the primary antibody (monoclonal mouse anti-NeuN serum [1:5000, Chemicon, Chandlers Ford, UK] in 1% normal horse serum, 1% BSA and 0.3% Triton X-100 in PBS), washed twice in PBS, and incubated for 2 h at room temperature in biotinylated horse antimouse serum (Vector Laboratories) (1:1000 in 1% BSA and 0.3% Triton X-100 in PBS). After further rinsing in PBS, they were placed for 2 h in avidin–biotin–horseradish peroxidase complex (1:200, ABC-Elite, Vector Laboratories) in PBS. After two further rinses in PBS, they were placed in a chromagen solution (0.05% diaminobenzidine [Sigma–Aldrich] and 0.01% H_2_O_2_ [Sigma–Aldrich]) for 5 min. The reaction was observed visually and stopped by rinsing in PBS. The sections were floated on to chrome-gelatine-coated slides and mounted with DPX.

An investigator who was blind to the behavioural results performed the microscopic examination. Drawings of the area of the lesions were superimposed on the appropriate coronal sections in the stereotaxic atlas of Paxinos and Watson [34].

### 2.6 Data analysis

Data from 5 of the 60 rats were discarded. Histological examination revealed two rats with misplaced excitotoxin-induced lesions; one rat in the disconnection + callosotomy group was discarded because of a failed callosotomy. Data from two further rats were discarded because they showed persistent exclusive responding on one lever. This left 13 rats in the sham-lesioned group, 14 in the disconnection group, 15 in the callosotomy group and 13 in the disconnection + callosotomy group.

#### 2.6.1 Preference functions and linear indifference functions

For each rat, the percentage choice of lever B in the free-choice trials (%*B*) was computed for each block of trials from the pooled data from the last 10 sessions of each phase of the experiment. Plots of %*B vs. d*_B_ were derived for each rat, and the indifference delay (*d*_B(50)_: the value of *d*_B_ corresponding to %*B* = 50%) was estimated by linear interpolation between the two delays which fell on either side of %*B* = 50% (*i* and *j*) using the formula: *d*_B(50)_ = *d*_B(*i*)_ + ([*d*_B(*j*)_-*d*_B(*i*)_].[%*B*_*i*_ − 50]/[%*B*_*i*_ − %*B*_*j*_]) [39]. Plots of *d*_B(50)_ *vs*. *d*_A_ were obtained for each rat, and linear functions were fitted by the method of least squares; goodness of fit was expressed as *r*^*2*^, the proportion of the data variance accounted for by the fitted function. The slope and intercept of the linear indifference functions were analysed by two-factor ANOVA (presence/absence of disconnection × presence/absence of callosotomy) followed by multiple comparisons of the lesioned groups with the sham-lesioned group using Dunnett's test. Linear indifference functions (*d*_B(50)_ *vs. d*_A_) were also derived for the group mean data. The slopes and elevations of these functions were analysed by one-factor ANOVA (group) followed by multiple comparisons of the lesioned groups with the sham-lesioned group using Dunnett's test, as described by Zar [43]: the slopes were first analysed, and in the absence of significant between-group variation in slope, a common weighted slope value was adopted in order to make comparisons among the elevations. (‘Elevation’ refers to the *y*-axis location of the function taking the range of observed data into account, whereas ‘intercept’ refers to the intersection of the function with the *y*-axis location [43].)

#### 2.6.2 Psychophysical analysis of preference functions

Logistic functions were fitted to the group mean %*B* data and the %*B* data from each rat in each phase of the experiment: %*B* = 100/(1 + [*d*_B_/*d*_B(50)_]^*ɛ*^). This function defines a descending sigmoid curve which is symmetrical in semi-logarithmic co-ordinates; *d*_B(50)_ and *ɛ* are parameters, *d*_B(50)_ being the point of intersection of the logistic curve with the indifference line, and *ɛ* being the slope of the function. These parameters were used to derive the limen ([*d*_B(25)_ − *d*_B(75)_]/2, where *d*_B(25)_ and *d*_B(75)_ are the estimated values of *d*_B_ corresponding to %*B* = 25 and %*B* = 75, respectively), and the index of relative precision, the Weber fraction, was defined as limen/*d*_B(50)_. The Weber fraction was subjected to repeated-measures ANOVA (phase); as no significant effect of phase was revealed, the Weber fractions were averaged across phases and subjected to two-factor ANOVA (presence/absence of disconnection × presence/absence of callosotomy), as described above.

## 3 Results

### 3.1 Behavioural data

#### 3.1.1 Preference functions and linear indifference functions

**Fig. 1:**
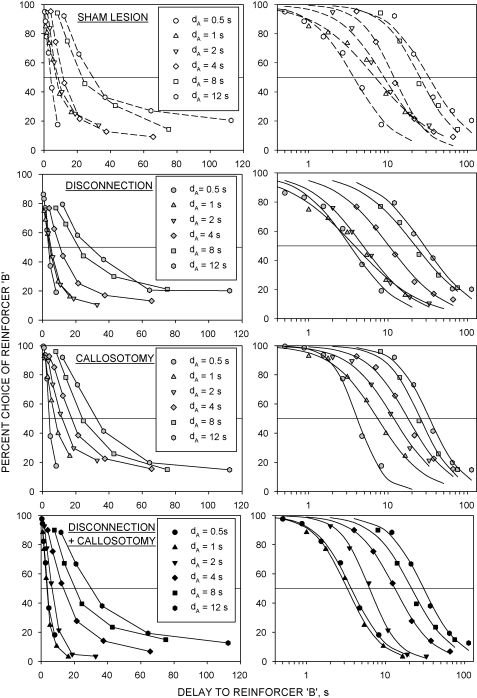
Group mean data from the sham-lesioned (*top row*), disconnection (*second row*), callosotomy (*third row*) and disconnection + callosotomy (*bottom row*) groups. *Left-hand panels* show preference functions (percent responding on lever B, %*B*, *vs.* delay to the larger of the two reinforcers after a response on B, *d*_B_, s). Each set of points shows data collected from one phase of the experiment, in which the delay to the smaller reinforcer (*d*_A_) was set at the value indicated (see inset). The horizontal reference line denotes indifference (%*B* = 50). The intersection between each preference function and the indifference level denotes the indifference delay (*d*_B(50)_) for that phase. *Right-hand panels* show transformations of the preference functions with *d*_B_ (s) on a logarithmic scale, and fitted logistic psychophysical functions, for each value of *d*_A_.

Preference functions (%*B vs. d*_B_) derived for the four groups in all six phases of the experiment are shown in Fig. 1 (left-hand graphs). In all four groups, preference for lever B declined as a function of the delay to reinforcer B (*d*_B_). The horizontal lines in the graphs show the indifference level (i.e., %*B* = 50); the value of *d*_B_ at which the preference function crossed this level (i.e. *d*_B(50)_) increased as a function of increasing values of *d*_A_, reflecting a progressive rightward displacement of the curve.

**Fig. 2:**
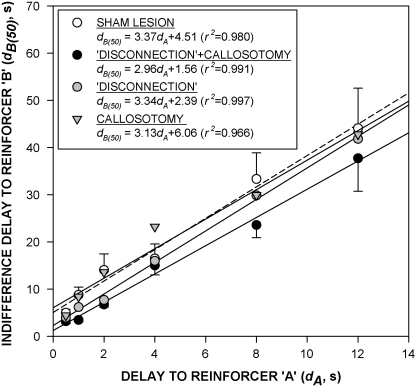
Linear indifference functions obtained for the sham-lesioned (*white circles*), disconnection (*grey circles*), callosotomy (*grey triangles*) and disconnection + callosotomy (*black circles*) groups. *Ordinate*: indifference delay to the larger reinforcer (*d*_B(50)_, s); *abscissa*: imposed delay to the smaller reinforcer (*d*_A_, s). Points show group mean data; vertical bars indicate S.E.M.s for the sham-lesioned and disconnection + callosotomy groups; lines are best-fit linear functions (see inset for the equations and goodness-of-fit of the fitted functions).

Fig. 2 shows indifference functions (*d*_B(50)_ *vs. d*_A_) for the group mean data. In each group, the linear function accounted for more than 96% of the variance of the group mean data (*r*^*2*^ > 0.96). Comparisons were made between the function derived for each of the lesioned groups and the function derived for the sham-lesioned group [43]. An initial test on the homogeneity of the slopes indicated that there was no significant effect of group upon the slope [*F*(3,20) = 1.8, *P* > 0.05], and the common (weighted) slope (3.16) was therefore adopted in statistical comparisons of the elevations of the functions. There was a significant effect of group on the elevation [*F*(3,23) = 24.4, *P* < 0.01]. Multiple comparisons (Dunnett's test) showed that the elevations of the functions derived for the disconnection and disconnection + callosotomy groups differed significantly from that of the sham-lesioned group [*t*(9) = 4.0, *P* < 0.05, and *t*(9) = 5.2, *P* < 0.01, respectively], whereas there was no significant difference between the elevations of the callosotomy and sham-lesioned groups [*t*(9) = 0.2, *P* > 0.3].

**Fig. 3:**
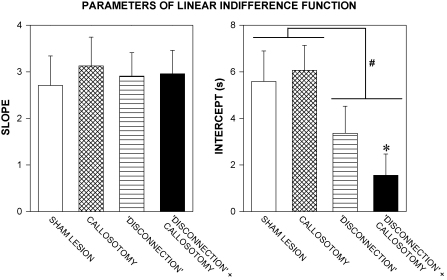
Parameters of the linear indifference functions from individual rats in the sham-lesioned (*unshaded*), callosotomy (*cross-hatched*), disconnection (*horizontally shaded*) and disconnection + callosotomy (*black*) groups. Columns show group mean values, vertical bars indicate S.E.M.s. *Left-hand panel*: slope of linear function; *right-hand panel*: intercept of linear function. Significant main effect of disconnection lesion: ^#^*P* < 0.05; significant difference from sham-lesioned group: *P < 0.05 (see text for details).

Linear indifference functions were also fitted to the data from the individual rats. The group mean values (+S.E.M.) of the slope and intercept of the function are shown in Fig. 3. A two-factor analysis of variance (callosotomy × disconnection) showed no significant effect of either factor nor any significant interaction on the slope [all *F*s < 1]. There was a significant effect of the disconnection lesion on the intercept [*F*(1,52) = 8.7, *P* < 0.005], but no significant effect of the callosotomy [*F* < 1] and no significant interaction [*F* < 1]. Multiple comparisons with the sham-lesioned group (Dunnett's test) showed that only the disconnection + callosotomy group differed significantly from the sham-lesioned group. The goodness of fit of the linear function did not vary significantly among the groups [*F* < 1]; the function accounted for >86% of the data variance for individual rats in all groups [*r*^*2*^ = 0.865 ± 0.022].

#### 3.1.2 Psychophysical analysis of preference functions

The logistic psychometric functions derived for the group mean data (Fig. 1, right-hand panels) accounted for 95% of the data variance (*r*^*2*^ > 0.95) in all cases. The logistic function could be fitted to 311 of the 330 preference functions obtained for the individual rats in the six phases of the experiment (94.2%); functions could be fitted to the data from all six phases in 48 of the 55 rats. Analysis of variance (disconnection × callosotomy) indicated that the goodness of fit was not significantly affected by either the callosotomy [*F* < 1] or the disconnection lesion [*F*(1,51) = 1.2, *P* > 0.2], and there was no significant interaction [*F* < 1]. The overall mean (±S.E.M.) value of *r*^2^ derived from all rats in all phases of the experiment was 0.966 ± 0.005. There was good agreement between the values of *d*_B(50)_ derived from the logistic functions (*d*_B(50)logist._) and those derived by linear interpolation (*d*_B(50)interp._). The slope of the regression of *d*_B(50)logist._ *vs. d*_B(50)interp._ (1.02 ± 0.01) did not deviate significantly from unity, and the intercept (−0.16 ± 0.31) did not deviate significantly from zero; the correlation (*r*) between the two estimates was 0.971.

**Fig. 4:**
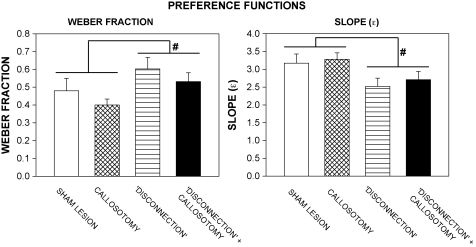
Weber fraction (*left-hand panel*) and slope parameter (*right-hand panel*) derived from psychophysical analysis of preference functions (see right-hand panels in Fig. 1). See text for derivation of the Weber fraction. Columns show group mean data; vertical bars indicate S.E.M.s; conventions as in Fig. 3.

The Weber fraction derived from the logistic function was not systematically related to the value of *d*_A_. A single-factor analysis of variance with repeated measures (incorporating data from the 48 rats that generated Weber fractions from all 6 phases) showed no significant effect of phase [*F*(5,235) = 1.9, *P* > 0.05]. The Weber fraction was therefore averaged across phases for each rat; the group mean values (+S.E.M.) are shown in Fig. 4 (left-hand histogram). Analysis of variance (callosotomy × disconnection) revealed a significant main effect of the disconnection lesion [*F*(1,51) = 6.6, *P* < 0.05], but no significant effect of the callosotomy [*F*(1,51) = 2.7, *P* > 0.1] and no significant interaction [*F* < 1]. Multiple comparisons (Dunnett's test) did not reveal any significant differences between the lesioned groups and the sham-lesioned group.

The group mean values of the slope of the logistic functions (*ɛ*) (+S.E.M.) are shown in Fig. 4 (right-hand histogram). Analysis of variance (callosotomy × disconnection) showed a significant main effect of the disconnection lesion [*F*(1,51) = 7.2, *P* < 0.05], but no significant main effect of the callosotomy [*F* < 1] and no significant interaction [*F* < 1]. None of the lesioned groups differed significantly from the sham-lesioned group (Dunnett's test).

### 3.2 Histology

**Fig. 5:**
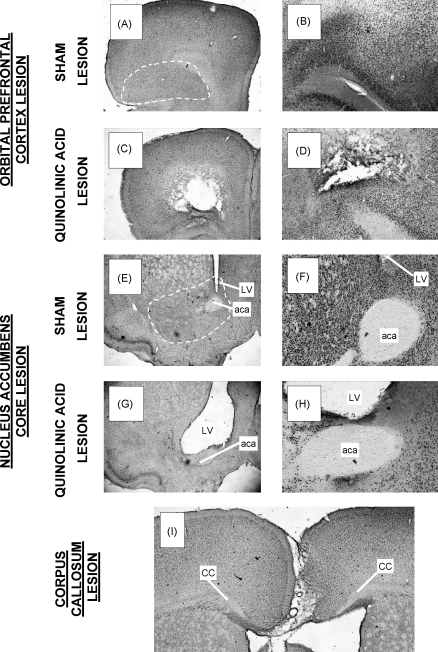
Photomicrographs showing examples of the lesions. (A–D) *OPFC*. (A) cresyl violet stained coronal section from a sham-lesioned rat; (B) section stained for NeuN. (C and D) corresponding sections showing quinolinic acid-induced lesion of the OPFC. Note the area of gliosis and neuronal loss in the OPFC. (E–H) *Nucleus accumbens core* (*AcbC*). (E and F): cresyl violet- and NeuN-stained sections from a sham-lesioned rat. (G and H) corresponding sections showing quinolinic acid-induced lesion of the AcbC. Note ventricular dilatation and neuronal loss. White broken lines in (A) and (E) show approximate extent of the OPFC and AcbC in the sham-lesioned rat. (I) *Callosotomy*: cresyl violet-stained section from a lesioned rat. Note the destruction of the corpus callosum, with attendant ventricular dilatation and damage to mesial surface of overlying cortex. LV: lateral ventricle; aca: anterior commissure (anterior portion); CC: corpus callosum.

Examples of the lesions are shown in Fig. 5. *OPFC*: injection of quinolinic acid into the OPFC resulted in gliosis and atrophy of the ventral and lateral orbital regions. There was some damage to the medial prefrontal cortex (medial orbital, infralimbic and prelimbic cortices) in some rats. The AP extent of the lesion was from about +3.2 to +4.5; in no case did the lesion extend caudally as far as the anterior margin of the nucleus accumbens. *AcbC*: coronal sections showed ventricular dilatation and atrophy in the ventral striatal area. The NeuN labelled sections showed that there was extensive neuronal loss in the area of the AcbC of all lesioned animals, with some neuronal loss in the ventral and medial portions of the caudate-putamen in some animals; the shell region of the nucleus accumbens was essentially spared. *Callosotomy*: the corpus callosum was completely severed between AP +0.5 to +1.6 in most animals. There was some sparing of the corpus callosum posteriorly (caudal to AP +0.5), possibly reflecting the curvature of the leucotome. Destruction of the callosum was generally accompanied by some ventricular dilatation in the vicinity of the lesion, and the mesial surfaces of the cortex overlying the lesion showed some damage.

## 4 Discussion

Injections of quinolinic acid into the OPFC and AcbC produced lesions of similar extent to those seen in previous experiments in which excitotoxins have been used to lesion these structures [*OPFC*: 13, 23, 25, 26, and 42; *AcbC*: 1, 2, 3, 5, 6, and 35]. In the case of the OPFC lesion, the main area of damage included the ventral and lateral orbital (VO and LO) regions. There was also some intrusion into the medial orbital (MO), infralimbic (IL) and prelimbic (PrL) regions in some animals. According to the subregional classification recommended by Uylings and van Eden [40] and Kesner [22], the lesion embraced the ventral (VO) and lateral OPFC (LO), with some involvement of the medial PFC (MO, IL and PrL). In the case of the AcbC lesion, the area of destruction was mainly restricted to the target structure. Some additional damage was inflicted to the ventral portion of the caudate-putamen in some rats; and in some cases NeuN staining revealed a band of neuronal loss in the medial caudate-putamen adjacent to the lateral ventricle. The mesial shell region of the nucleus accumbens was spared. The callosotomy lesion was generally successful in severing the corpus callosum anterior to the caudal margin of the AcbC.

The discrete-trials schedule used in this study was an adaptation of the progressive delay schedule developed by Evenden and Ryan [10]. The rats in all four groups showed a progressive shift in preference from the larger to the smaller reinforcer as the delay to the larger of the two reinforcers (*d*_B_) was progressively increased across successive blocks of trials. This is consistent with previous studies that have used this schedule [2,6–8,10,11,23,24,26,33,42]. As in previous experiments [2,23,24,26], we used a geometric progression to determine the values of *d*_B_ in successive blocks of trials, thereby allowing the range of values of *d*_B_ to be adapted to the value of *d*_A_, which was systematically manipulated across the six phases of the experiment. The resulting preference functions (relation between %*B* and *d*_B_; see Fig. 1) were used to compute the indifference delays to the larger reinforcer (*d*_B(50)_) in each phase. This measure formed the basis of the linear indifference functions (see below).

As in previous studies employing Evenden and Ryan's [11] protocol [2,6–8,10,11,23,24,26,33,42], the preference functions seen in this experiment were characterized by a gradual reduction of %*B* as a function of *d*_B_. As noted by Bezzina et al. [2], this is apparently inconsistent with models of inter-temporal choice that are based on the computation of hypothetical ‘values’ of reinforcers. Such models generally assume that organisms should invariably select the more highly valued of two mutually exclusive reinforcers [18,19], leading to the prediction that the ideal preference function should be a step function, %*B* falling precipitously from near 100% to near 0% around the point at which *V*_A_ = *V*_B_. Bezzina et al. [2] proposed that the gradual decline in preference generated by progressive delay schedules may represent a discrimination gradient for reinforcer value, and adopted a standard psychophysical approach to analyse the preference functions, an analytical approach that was also used in the present study. As in Bezzina et al.'s [2] experiment, a two-parameter logistic function adequately described the group mean preference functions (Fig. 1, right-hand panels) and more than 90% of the preference functions obtained from individual rats. The two parameters of this function define its slope (*ɛ*) and its locus on the abscissa (*d*_B(50)_). Combination of *ɛ* and *d*_B(50)_ allows computation of the Weber fraction, the traditional measure of the relative precision of discrimination [15,16,20,28]. The data shown in Fig. 4 indicate that *ɛ* tended to be lower and the Weber fraction higher in the disconnection-lesioned and disconnection + callosotomy groups than in the sham-lesioned group. This resembles Bezzina et al.'s [2] finding with bilateral AcbC lesions, which induced a robust increase in the Weber fraction, consistent with an impairment of discriminative precision. It appears that functional disconnection of the OPFC from the AcbC had a similar effect on value discrimination as destruction of the AcbC.

The locus of the preference function is defined by *d*_B(50)_, the delay to reinforcer B corresponding to indifference between the two reinforcers. According to hyperbolic models of inter-temporal choice [19,29], indifference implies equality of the values of the two reinforcers, which provides the basis for deriving the linear function expressed by Eq. (2). This equation offers a means of distinguishing between changes in inter-temporal choice behaviour brought about by effects on the hypothetical processes of delay discounting (*K*) and magnitude discounting (*Q*). The slope of the linear function reflects the physical magnitudes of the two reinforcers (*q*_A_ and *q*_B_) and *Q*; a change in slope therefore implies a change in *Q*. The intercept of the function is influenced jointly by *Q* and *K*. Therefore, while an increase in *Q* causes an increase in both the slope and the intercept, an increase in *K* simply diminishes the intercept. If both parameters are increased, *Q*'s effect on the intercept is countered by the change in *K*, whereas its impact on the slope remains unaltered [19].

In the present experiment, the indifference functions of the four groups did not differ significantly in terms of slope; however, there were significant between-group differences in the intercept. The disconnection-lesioned group showed a somewhat lower intercept than the sham-lesioned group. This effect was statistically significant when the functions fitted to the group mean data were compared; however it failed to reach significance when the functions fitted to the data from individual rats in the two groups were compared. A more robust effect on the intercept was seen in the case of the disconnection + callosotomy group, whose intercept was significantly lower than that of the sham-lesioned group, in the case of the functions fitted to the group mean data and also the functions fitted to the individual-subject data. The callosotomy had no significant effect on the intercept. These results indicate that functional disconnection of the AcbC from the OPFC selectively altered the delay discounting parameter, *K*. The lower intercept seen in the disconnection + callosotomy group implies a higher value of *K*, in other words, a higher rate of delay discounting, than that of the sham-lesioned group. The fact that callosotomy alone had no discernable effect on performance suggests that ipsilateral cortifugal fibres may be mainly responsible for the functional connection between the OPFC and AcbC; however, the fact that the combined disconnection + callosotomy lesion had a more robust effect on inter-temporal choice than the conventional lesion suggests that inter-hemispheric connections may also make a subsidiary contribution. The existence of inter-hemispheric cortico-striatal connections has been known for some time [32], although their functional importance has only recently been established. Dunnett and his colleagues [9,41] recently found that a combined disconnection + callosotomy lesion was needed to effect complete functional disconnection of the prefrontal cortex and dorsal striatum in the case of delayed alternation performance.

Previous experiments have examined the effects of selective lesions of the OPFC and AcbC on inter-temporal choice behaviour. Kheramin et al. [23,26] found that bilateral destruction of the OPFC increased the slope of the linear indifference function without altering the intercept, an effect that was attributed to an increase in the rate of delay discounting combined with increased sensitivity to relative reinforcer size (see also [25]). Cardinal et al. [6,8] first reported that bilateral lesions of the AcbC rendered rats more ‘intolerant’ to delay of reinforcement. This finding was recently confirmed and extended by Bezzina et al. [2], using the indifference-equation approach; these authors reported that destruction of the AcbC lowered the intercept of the linear indifference function without significantly altering the slope, implying an increase in the rate of delay discounting. These findings suggest that both the OPFC and AcbC may contribute to delay discounting. The present results further suggest that delay discounting may be regulated by an integrated mechanism that involves both these structures, and that the integrity of both structures and their connecting fibres may be important for the effective control of behaviour by delayed reinforcers. Interestingly, the present results provide no evidence for an involvement of OPFC–AcbC connections in regulating sensitivity to reinforcer size. This suggests that while the OPFC may integrate information on multiple features of reinforcers, including both size and delay [37,38], its role in delay discounting may be more specifically related to its connections with the AcbC. Data consistent with this notion have recently been obtained using the progressive ratio schedule. Mathematical analysis of performance on this schedule [27] yields a quantitative index of instantaneous reinforcer value that is sensitive to variations of reinforcer size [4,36]. This index has been found to be reduced by lesions of the OPFC [25] but not by lesions of the AcbC [3], suggesting that the OPFC, but not the AcbC, may be involved in determining sensitivity to reinforcer size.
